# Functional genomics analysis of Phelan-McDermid syndrome 22q13 region during human neurodevelopment

**DOI:** 10.1371/journal.pone.0213921

**Published:** 2019-03-15

**Authors:** Catherine A. Ziats, Luke P. Grosvenor, Sara M. Sarasua, Audrey E. Thurm, Susan E. Swedo, Ahmed Mahfouz, Owen M. Rennert, Mark N. Ziats

**Affiliations:** 1 Division of Intramural Research, National Institute of Child Health and Human Development, National Institutes of Health, Bethesda, Maryland, United States of America; 2 Pediatrics and Developmental Neuroscience Branch, National Institute of Mental Health, National Institutes of Health, Bethesda, Maryland, United States of America; 3 Simons Foundation Autism Research Initiative, New York, New York, United States of America; 4 School of Nursing, Clemson University, Clemson, South Carolina, United States of America; 5 Delft Bioinformatics Lab, Delft University of Technology, Delft, The Netherlands; 6 Department of Internal Medicine, Michigan Medicine, Ann Arbor, Michigan, United States of America; Universitaire Ziekenhuizen Leuven, BELGIUM

## Abstract

Phelan-McDermid syndrome (PMS) is a neurodevelopmental disorder characterized by varying degrees of intellectual disability, severely delayed language development and specific facial features, and is caused by a deletion within chromosome 22q13.3. *SHANK3*, which is located at the terminal end of this region, has been repeatedly implicated in other neurodevelopmental disorders and deletion of this gene specifically is thought to cause much of the neurologic symptoms characteristic of PMS. However, it is still unclear to what extent *SHANK3* deletions contribute to the PMS phenotype, and what other genes nearby are causal to the neurologic disease. In an effort to better understand the functional landscape of the PMS region during normal neurodevelopment, we assessed RNA-sequencing (RNA-seq) expression data collected from post-mortem brain tissue from developmentally normal subjects over the course of prenatal to adolescent age and analyzed expression changes of 65 genes on 22q13. We found that the majority of genes within this region were expressed in the brain, with *ATNX10*, *MLC1*, *MAPK8IP2*, and *SULT4A1* having the highest overall expression. Analysis of the temporal profiles of the highest expressed genes revealed a trend towards peak expression during the early post-natal period, followed by a drop in expression later in development. Spatial analysis revealed significant region specific differences in the expression of *SHANK3*, *MAPK8IP2*, and *SULT4A1*. Region specific expression over time revealed a consistently unique gene expression profile within the cerebellum, providing evidence for a distinct developmental program within this region. Exon-specific expression of *SHANK3* showed higher expression within exons contributing to known brain specific functional isoforms. Overall, we provide an updated roadmap of the PMS region, implicating several genes and time periods as important during neurodevelopment, with the hope that this information can help us better understand the phenotypic heterogeneity of PMS.

## Introduction

Phelan-McDermid Syndrome (PMS) is a rare genetic disorder, characterized predominately by a neurodevelopmental phenotype. The disorder is typically considered in children with varying degrees of intellectual disability, severely delayed language development, and a typical facies that may include dolichocephaly, full brows, and a flat midface among other features [1–4]. However, the phenotypic spectrum is wide and thus, the diagnosis is made with laboratory testing to establish a deletion at chromosome 22q13.3. Relatively little is known about the relationship between genotype and corresponding phenotype in PMS, especially as it relates to the neurologic manifestations of the disease. There is evidence to suggest that larger deletions on 22q13 tend to be associated with a more severe phenotype [5–10], however the relationship is not consistent, as patients with similar size deletions can have varied disease presentations [1, 10–12]. *SHANK3*, located at the terminal end of the Phelan-McDermid region, has been repeatedly implicated in the pathogenesis of neurodevelopmental disorders such as autism and intellectual disability [13], as well as psychiatric diseases such as schizophrenia [14, 15] and bipolar disorder [16]; its protein product known to play an important role in synaptic plasticity and maintenance of long term potentiation [17, 18]. While several groups have implicated *SHANK3* as the causative gene for the neurologic deficits in Phelan-McDermid patients [7–9, 12, 19–22], a deletion or variant in this gene is not considered necessary for diagnosis [11, 23].

It is likely that other genes also influence the neurologic sequelae of PMS as patients with similar neurologic phenotypes and 22q13 deletions proximal to *SHANK3* have been reported [11, 24–28]. *MAPK8IP2*, located about 70 kb proximal to *SHANK3*, and frequently co-deleted in PMS, is one such gene that has been implicated due to the high expressivity of the protein product in the brain at the post synaptic density, and studies showing mice with absence of *MAPK8IP2* have abnormal dendritic morphology, as well as motor and cognitive deficits [29]. Despite this work, it is still unclear how deletions in *SHANK3* and *MAPK8IP2* specifically, contribute to the neurologic phenotype of PMS and what other genes or combination of genes contribute to the complex and varied symptomology. In an attempt to better characterize the functional landscape of the 22q13 region, we assessed the expression patterns of genes mapped within the Phelan-McDermid region in neurologically normal human brain samples over developmental time. Our results provide insight into the importance of 22q13 during normal human neurodevelopment and suggest other genes, with similar expression patterns to *MAPK8IP2* and *SHANK3*, that may contribute the neurologic phenotype in PMS.

## Materials and methods

### Developing human brain transcriptome data

The *BrainSpan* transcriptional atlas was downloaded from http://www.brainspan.org, where specific details regarding tissue acquisition and processing can be found [30, 31]. The online dataset contains next-generation RNA-sequencing data obtained from 41 donors ranging from pre-natal development (8 postconception weeks) to adulthood (40 years of age). All donor brains came from patients who until time of death were considered to be neurologically and developmentally normal. For each donor, sequencing data is available from several distinct brain regions (maximum 16 regions), however several donors have missing data from certain brain regions, and donors with more than 6 regions missing were excluded. For those donors with six or less missing brain regions, the missing data was imputed using a nearest neighbor approach, as we previously described [32]. After excluding the donors with missing data, a total of 30 brains with sequencing data for 16 brain regions were analyzed: cerebellar cortex, medial dorsal nucleus of thalamus, striatum, amygdala, hippocampus, and 11 regions of the neocortex. For comparative purposes, we binned the 30 brain samples into 7 discrete developmental time periods (16 post-conception weeks– 17 post-conception weeks, 19 post-conception weeks– 24 post-conception weeks, 4 months– 1 year, 2 years– 4 years, 8 years– 13 years, 15 years– 21 years, 23 years– 40 years) as has been previously used to analyze this dataset [32–35]. The resulting dataset consisted of RNA-sequencing expression values for 524 tissue samples given in units of reads per kilobase of exon model per million mapped reads (RPKM) [36].

### 22q13 gene set

The UCSC Genome Browser, genome assembly GRCh38/hg38 released December 2013 (https://genome.ucsc.edu/)), was used to identify genes within the PMS 22q13 region (coordinates: hg 19 chr22: 37,600,001–51,304,566) [37]. After exclusion of genes and other RNA species not present in the *BrainSpan* dataset, 65 protein-coding genes remained for inclusion in the final dataset (S3 Table). Average RPKM per gene was calculated for all developmental time periods and all brain regions. An extensive literature search was performed on all genes with at least one read greater than five RPKM (S1 and S2 Tables), which we considered to be biologically significant brain expression [32]. The four genes with the highest expression were further analyzed. The expression profile of *SHANK3* was also analyzed given the importance of this gene in the neurologic manifestations of disease.

### Analysis of highly expressed genes

Total average expression per gene per developmental time period was calculated and one-way analysis of variance (ANOVA) were run to assess significance. Pre-natal and post-natal expression were compared using student t-test analysis, with p<0.05 set as significant. Region specific expression (total and over developmental time) was also calculated and ANOVA analyses were performed to test difference between region. Expression was assessed specifically in the amygdala, cerebellar cortex, hippocampus, dorsolateral prefrontal cortex, ventrolateral prefrontal cortex, and striatum as these regions have been consistently implicated in neurodevelopmental disorders [38–42]. Additionally, for the *SHANK3* gene, average RPKM was calculated for each exon and compared.

## Results

### Expression of genes on 22q13 reveals subset with high brain tissue specific expression

**Fig 1:**
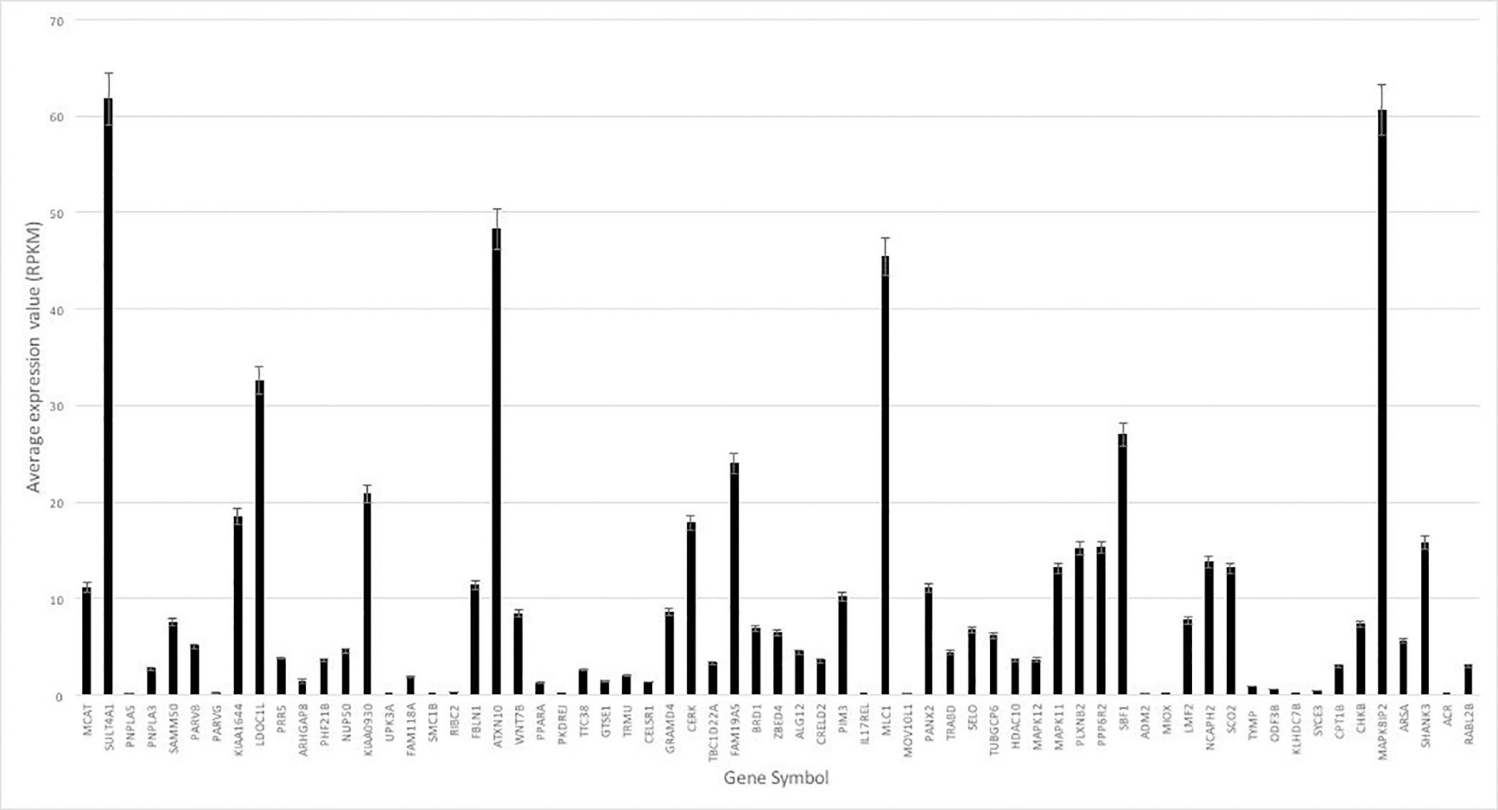
Average expression of 22q13 genes. The average expression value for each of the 65 genes on 22q13 is shown, arranged from most proximal (left) to most distal (right) on chromosome. Error bars represent the standard error of the mean.

**Fig 2:**
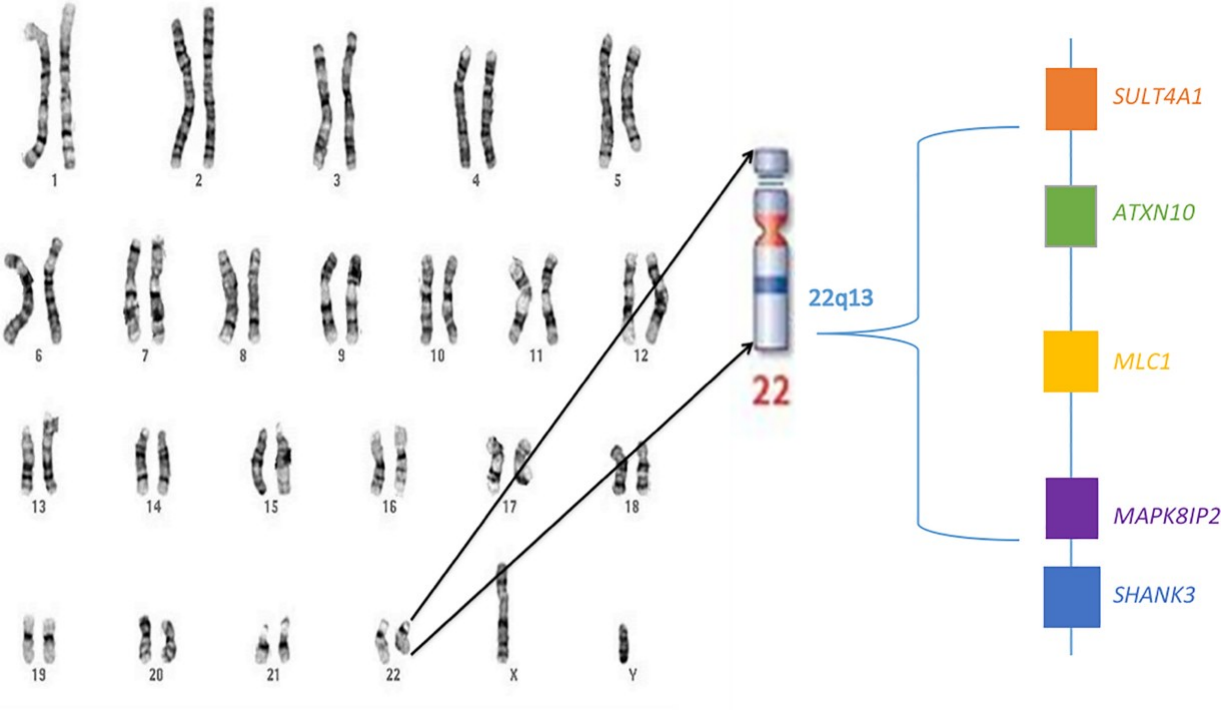
22q13 region. The location of the highest expressed genes in relation to *SHANK3* is shown.

The average expression value (in RPKM) for each of the 65 genes used in the analysis was calculated over all developmental time periods and all brain regions assessed (Fig 1). Six (9%) of the genes did not have at least one read greater than five RPKM, and gene expression of those genes was interpreted as noise, as reported previously [32]. The mean total expression for *SHANK3* was 15.82 RPKM (standard deviation (SD) = 12.10) vs 9.59 (SD = 13.60) for the entire set of 65 genes over all time periods and brain regions. Four genes, *ATXN10* (mean (M) = 48.24, SD = 17.30), *MAPK8IP2* (M = 60.61, SD = 26.40), *MLC1* (M = 45.40, SD = 56.38), and *SULT4A1* (M = 61.81, SD = 50.15), had average expression at least two standard deviations above the average expression for the entire gene set (Figs 1 and 2).

### Expression of highly expressed genes reveals shared temporal profile with *SHANK3*

**Fig 3:**
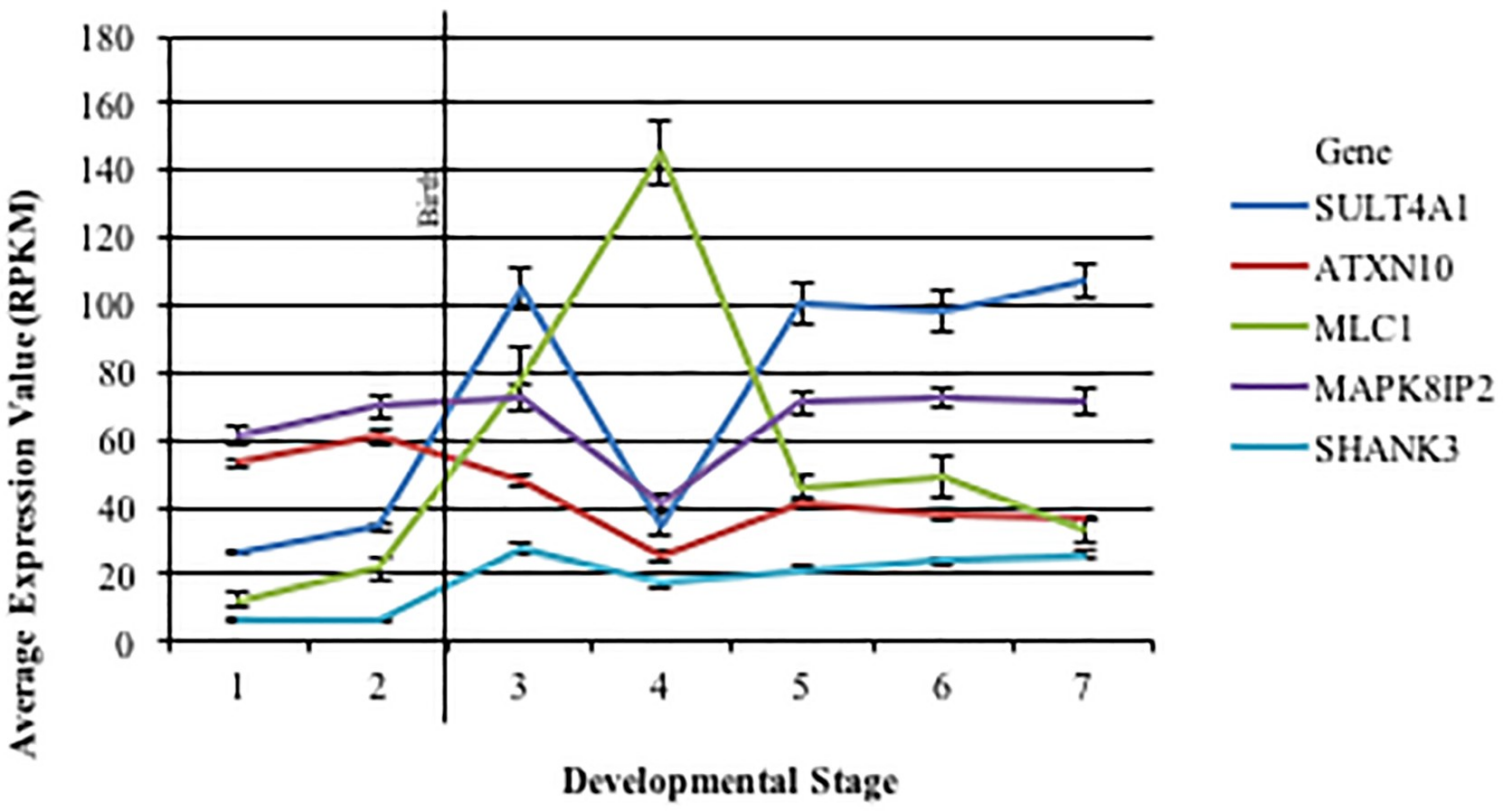
Average expression across developmental time. The average expression of each gene assessed across seven developmental stages is shown (**1**: 16 pcw– 17 pcw, **2**: 19 pcw– 24 pcw, **3**: 4 mos– 1 yr, **4**: 2 yrs– 4 yrs, **5**:8 yrs– 13 yrs, **6**: 15 yrs– 21 yrs, **7**: 23 yrs– 40 yrs). Error bars represent the standard error of the mean. pcw = post-conception years; mos = months; yrs = years.

Average expression of *ATXN10*, *MAPK8IP2*, *MLC1*, *SULT4A1* and *SHANK3*, incorporating all brain regions, over developmental time was calculated (Fig 3). Overall the tendency was for a bimodal pattern, with highest expression during infancy and a decrease in expression over early childhood, followed by gradual increase and eventual plateau later in childhood to adulthood. Notably, *MLC1* and *ATXN10* expression did not follow a bimodal pattern, expression did not increase later in childhood. Additionally, *MLC1* showed a delay in peak expression, with highest expression being in early childhood, while *ATXN10* showed early peak expression, with highest expression during gestation.

One-way ANOVA test showed a significant main effect of developmental time on gene expression for all five genes assessed, p-value <0.05 (S4 Table). When pre-natal vs post-natal expression was compared, post-natal expression was found to be significantly higher than pre-natal expression for *SHANK3*, *MAPK8IP2*, and *SULT4A1*. Pre-natal expression was significantly higher than post-natal expression for *ATXN10*, while no significant difference between pre- and post-natal expression was found for *MLC1* (S4 Table).

### Expression of highly expressed genes reveals unique spatial profiles

**Fig 4:**
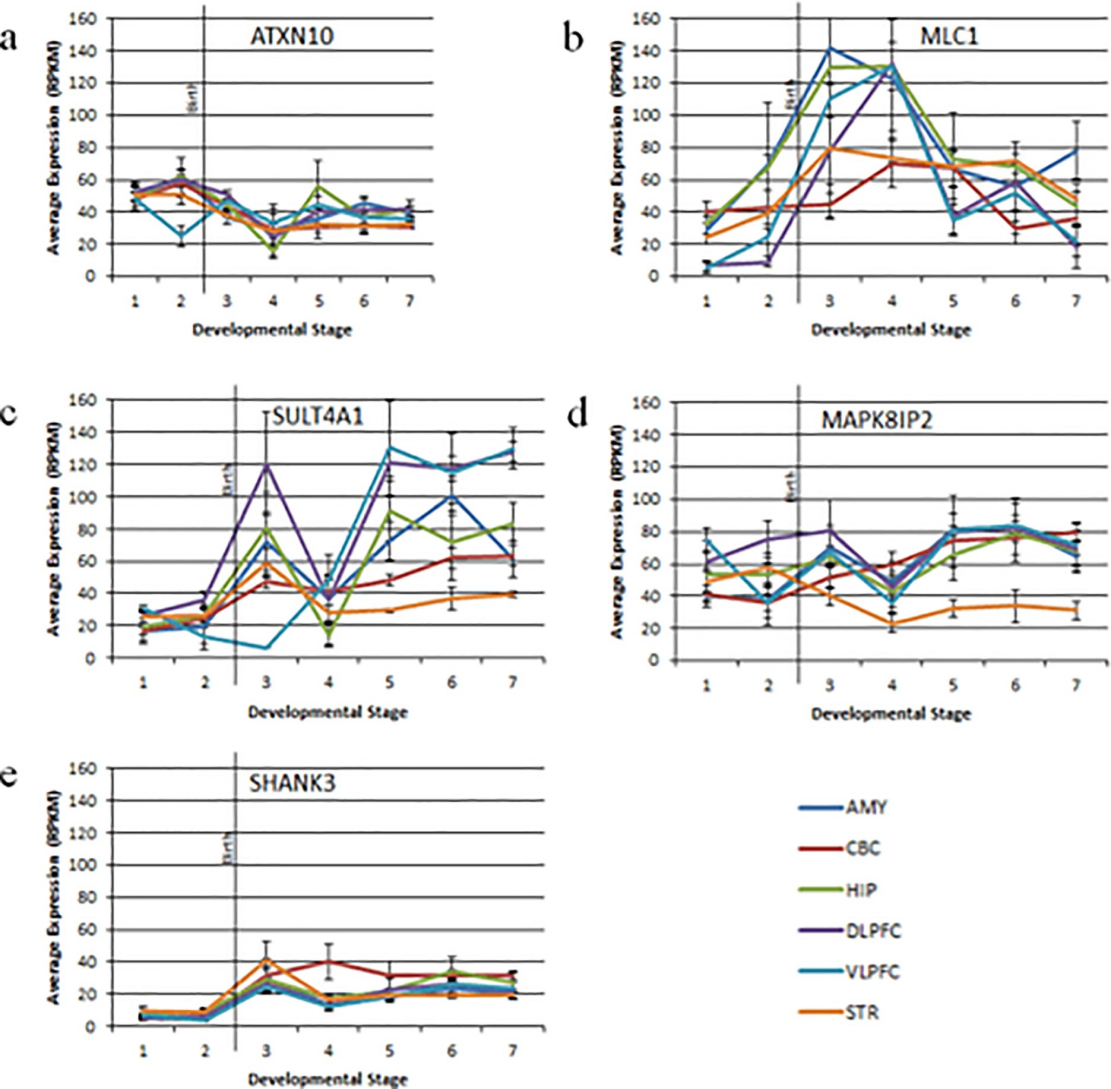
Average expression across time and space. The expression profile for each candidate gene across developmental time in six different brain regions (AMY, CBC, HIP, DLPFC, VLPFC, STR) is shown in a-e (a: *ATXN10*, b: *MLC1*, c: *SULT4A1*, d: *MAPK8IP2*, e: *SHANK3*). The black vertical line represents birth. Error bars represent the standard error of the mean. AMY = amygdala, CBC = cerebellum, HIP = hippocampus, DLPFC = dorsolateral prefrontal cortex, VLPFC = ventrolateral prefrontal cortex, STR = striatum.

Average brain region specific expression of the four highest expressed genes as well as *SHANK3* was analyzed (S5 Table). Expression of *SHANK3*, *MAPK8IP2*, and *SULT4A1* was significantly different between brain regions when all developmental time periods were included. *SHANK3* showed highest expression in the cerebellum, while *MAPK8IP2* and *SULT4A1* showed highest expression in the dorsolateral prefrontal cortex. We also assessed region specific expression over developmental time. Overall, spatial patterns of expression over time were largely consistent with average expression over time, although gene specific expression within the cerebellum did not seem to follow this trend (Fig 4).

### Expression of SHANK3 exons

**Fig 5:**
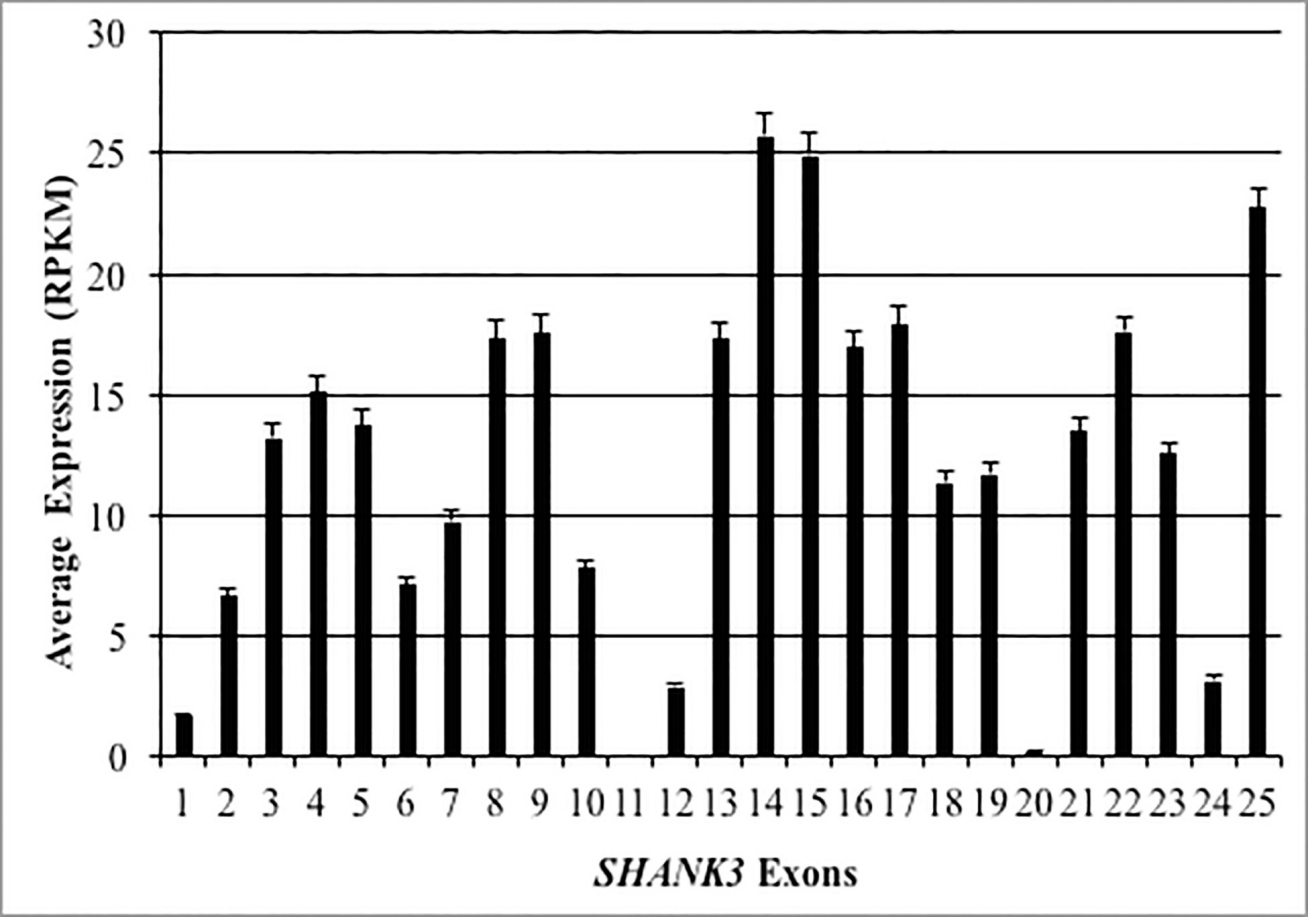
Average expression of *SHANK3* exons. The average expression of each individual *SHANK3* exon across all brain regions and developmental time periods is shown. Error bars represent the standard error of the mean.

*SHANK3* exon-specific average expression was calculated for all regions and all time periods (Fig 5). Notably, 5/25 exons (Exons 1, 11, 12, 20, and 24) did not reach five RPKM of average expression. The exons with the highest brain specific expression were 14, 15 and 25.

## Discussion

Patients with Phelan-McDermid Syndrome can present with a wide range of neurologic symptoms of varying severity, making clinical diagnosis difficult. Appropriate diagnosis relies upon molecular testing to confirm a deletion on 22q13. While evidence supports the role of *SHANK3* in the neuropathogenesis of the disease, it is unclear how other genes on 22q13 also contribute to the neurologic phenotype [7, 8, 10, 11, 21, 26, 28]. Traditional approaches have relied on rodent knock-out models to study the correlation between genotype and phenotype, however these studies are cumbersome and the subtle neurologic symptoms typical of PMS are not ideally suited for animal behavioral models [17, 18, 29]. More recently, candidate genes have been identified with detailed phenotype mapping, using array comparative genome hybridization data to correlate deletions of a specific location and size with clinical symptoms [11, 43]. While, this approach results in the identification of many candidate genes, in the absence of tissue specific data, the developmental context and significance of these genes in the pathology of human diseases remains unknown. In this manuscript we describe a complementary approach; we use gene expression data to examine the Phelan-McDermid region during normal development, which we show provides insight into the functional landscape of this region.

Our analysis of gene expression trends over developmental time revealed specific expression patterns of known functional significance. The gene *ATXN10* showed a pattern of increased gene expression during gestation and decreased expression after birth, while *SULT4A1*, *SHANK3*, and *MAPK8IP2* expression remained high throughout fetal life and infancy. Interestingly, large genome-wide transcriptome analyses have shown these distinct patterns of gene expression have functional significance, and specifically, enrich for genes related to axonal growth and synaptic function, respectively, highlighting the importance of the PMS region during normal neurodevelopment [44]. Notably, in a detailed review of 22q13.3 genes likely to be haploinsufficient, Mitz *et al*. also identified *SULT4A*, *MAPK8IP2*, and *ATXN10* as likely candidates for the PMS phenotype. [45].

Our analysis of gene expression trends over developmental time also provide insight into potential mechanisms of disease pathogenesis. For instance, the gene *MLC1* showed a delayed pattern of peak gene expression, with highest expression profiles during early childhood, a period typically characterized by decreased overall expression in the brain [44]. We can speculate that this pattern of expression may explain, in part, the childhood developmental regression typical of megalencephalic leukoencephalopathy (MLC), a disorder caused by variants in *MLC1* [46]. Moreover, as childhood developmental regression is described in up to 50% of PMS patients [47], future work may focus on further characterizing the relationship between *MLC1* deletions and regression symptoms in PMS patients.

Spatial analysis revealed that in most of the genes assessed, there was significantly different region-specific expression. This is likely a product of differences in function between brain regions, however it highlights the importance of spatial context for understanding normal neurodevelopment. Overall, region specific gene expression showed largely conserved trends in gene expression over developmental time, with the exception of the cerebellum. This finding however, is not unique to our study, as several groups have shown whole genome cerebellar expression to be distinct as compared to other brain regions [48, 49]. Additionally, while the cerebellum is consistently implicated in neurodevelopmental disorders and as having a role in higher cognitive function, how the unique developmental timeline of this region plays a role in the pathology of such diseases is still unknown [50, 51].

Given the importance of *SHANK3* in the pathogenesis of PMS and known complex cell type and region specific transcriptional regulation, we analyzed exon specific expression, which confirmed high brain-specific expression of known functional isoforms [52]. Exons encoding known functional domains that act at the post-synaptic density (PSD), notably ankryrin repeat domain (exons 4–7), the PSD protein/*Drosophila* disc large tumor suppressor/zonula occludens-1 protein (PDZ; exons 13–16), and the Homer binding domain (exon 21), were all highly expressed [17, 53, 54].

For this study we specifically chose to analyze the expression of genes within the PMS region with the highest overall gene expression however it is likely that genes with lower baseline expression also contribute to the pathophysiology of PMS, and we have reviewed these genes in the supplemental information (S1 and S2 Tables). For instance, the gene *ARSA*, which had a total average gene expression of 5.6 RPKM, encodes the enzyme arylsulfatase A and is situated near *SHANK3* at the terminal end of the chromosome 22q13.33, a region commonly in deleted in PMS [21]. However as variants in this gene lead to metachromatic leukodystrophy, a devastating disease characterized by severe neurologic symptoms including progressive mental deterioration, hypotonia, weakness and seizures, how exactly deletions in this gene may contribute to the neurological sequelae of PMS should be further explored [55].

Our manuscript is limited by our hypothesis generating approach. However, it is our intention that our data be used to draw insight into the developmental and spatial relationships of genes within the PMS region, which may then be used to test hypotheses using traditional functional studies. Additionally, while we focus on protein-coding genes specifically, non-coding RNAs (ncRNAs) are known to play an active role in transcriptional regulation during neurodevelopment and so understanding the dynamic expression of these genes in the PMS region and how they modulate the local transcriptional landscape will be important and deserves further study [34].

## Conclusion

The broad Phelan-McDermid phenotype encompasses a range of neurologic symptoms of varying severity and consequence; the etiology of which remains poorly understood. In this brief report we use gene expression data from normal controls to glean insight into the functional landscape of the 22q13.3 region. This work begins to show how the PMS region is involved in normal neurodevelopment, and specifically how dynamic temporal and spatial expression profiles may hint at gene function and mechanisms of disease, and moreover may be used to guide future functional studies.

## Supporting information

S1 Table Gene function and clinical phenotypes associated with the 31 protein-coding genes with average whole brain expression over 5 RPKM. (DOCX)

S2 Table Gene function and clinical phenotypes associated with the 18 protein-coding genes with more than one read > 5 RPKM but with overall average gene expression <5. Developmental time period when RPKM >5 also shown. (1: 16 pcw– 17 pcw, 2: 19 pcw– 24 pcw, 3: 4 mos– 1 yr, 4: 2 yrs– 4 yrs, 5:8 yrs– 13 yrs, 6: 15 yrs– 21 yrs, 7: 23 yrs– 40 yrs). (pcw = post-conception weeks, mos = months, yrs = years).
(DOCX)

S3 Table Expression of genes within 22q13 region. The average expression in RPKM and SD of each of the 65 protein coding genes within the PMS region is shown. Genes are ordered by most proximal to most distal on chromosome.
(DOCX)

S4 Table Expression data over developmental time. Average expression of each gene assessed by developmental stage shown. Results of ANOVA testing and t-test also displayed. (1: 16 pcw– 17 pcw, 2: 19 pcw– 24 pcw, 3: 4 mos– 1 yr, 4: 2 yrs– 4 yrs, 5:8 yrs– 13 yrs, 6: 15 yrs– 21 yrs, 7: 23 yrs– 40 yrs). (pcw = post-conception weeks, mos = months, yrs = years).
(DOCX)

S5 Table Expression data per region. Average brain region specific expression of each gene assessed is shown. Results of ANOVA testing is also displayed. Significant p-values are bolded. (AMY = amygdala, CBC = cerebellum, HIP = hippocampus, DLPFC = dorsolateral prefrontal cortex, VLPFC = ventrolateral prefrontal cortex, STR = striatum).
(DOCX)
