# Supplementary material for: Functional genomics analysis of Phelan-McDermid syndrome 22q13 region during human neurodevelopment
Source: PLoS One. 2019 Mar 15;14(3):e0213921. doi: 10.1371/journal.pone.0213921 (PMC6420160; doi:10.1371/journal.pone.0213921)
Supplement: S1 Table — (DOCX) [file pone.0213921.s001.docx]

**S1 Table.** Gene function and clinical phenotypes associated with the 31 protein-coding genes with average whole brain expression over 5 RPKM.

| **Gene symbol** | **OMIM number [1]** | **Function of protein product** | **associated disease** | **Associated neurologic phenotype** | **Other associated phenotype** | **References** |
| --- | --- | --- | --- | --- | --- | --- |
| *MCAT* | 614479 | Biosynthesis of fatty acids within mitochondria | Unknown | Unknown | male genital anomalies, short stature | [2, 3] |
| *SULT4A1* | 608359 | metabolism of endogenous chemicals, known expression in cerebellum, hypothalamus and cortex | Unknown | Speech abnormalities, absence of ASD, sedentary behavior | Male genital abnormalities | [3-6] |
| *SAMM50* | 612058 | Component of outer mitochondrial membrane | non-alcoholic fatty liver disease | Speech abnormalities, absence of ASD | Hair pulling, male genital abnormalities | [3, 7, 8] |
| *PARVB* | 608121 | Actin binding protein, known to interact with ARHGEF6 (associated with X-linked ID) | non-alcoholic fatty liver disease, ID | Speech abnormalities, absence of ASD | Hair pulling, male genital abnormalities, large hands | [3, 8, 9] |
| *KIAA1644* | n/a | Transmembrane protein with unknown function | Unknown | Speech abnormalities, absence of ASD | Hair pulling, male genital abnormalities, large hands | [3, 10] |
| *LDOC1L* | n/a | Unknown | Unknown | Speech abnormalities, absence of ASD | Hair pulling, male genital abnormalities, large hands, facial asymmetry | [3] |
| *KIAA0930* | n/a | Unknown | Unknown | Unknown | Unknown |  |
| *FBLN1* | 135820 | Extracellular matrix protein | Synpolydactyly-2 | Abnormal reflexes, speech abnormalities, absence of ASD, neonatal hypotonia | Metatarsal fusion, metacarpal fusion, synpolydactyly, tall stature, dysplastic toenails, facial asymmetry, large hands, hair pulling, male genital abnormalities | [3, 11, 12] |
| *ATXN10* | 61150 | Unknown | Spinocerebellar ataxia 10 | Seizures, cerebellar ataxia, neonatal hypotonia, abnormal reflexes, speech abnormalities, absence of ASD, macrocephaly | tall stature, dysplastic toenails, facial asymmetry, large hands, hair pulling, male genital abnormalities, late walking | [3, 6, 13] |
| *WNT7B* | 601967 | Member of WNT signaling family of molecules involved in dendrite formation and central nervous system endothelium | Unknown | neonatal hypotonia, speech abnormalities, absence of ASD, macrocephaly | dysplastic toenails, large hands, hair pulling, male genital abnormalities, late walking | [3, 14, 15] |
| *GRAMD4* | 613691 | Mitochondrial effector of E2F1-indiced apoptosis | Unknown | Macrocephaly, neonatal hypotonia, absence of ASD | Large hands, dysplastic toenails, late walking, male genital abnormalities | [3, 16] |
| *CERK* | 610307 | Converts ceramide to ceramide-1-phosphate (a sphingolipid metabolite). Implicated in cellular processes such as apoptosis, inflammation, phagocytosis, proliferation | Unknown | Macrocephaly, neonatal hypotonia, absence of ASD | Large hands, dysplastic toenails, late walking, male genital abnormalities | [3, 17] |
| *FAM19A5* | 617499 | Small secreted protein with unknown function | Methylation marking in high risk cervical cancer | Neonatal hypotonia | Late walking, dysplastic toenails, large hands | [3, 18, 19] |
| *BRD1* | 604589 | Scaffold protein involved in controlling gene expression of a number of chromatin-associated genes including genes implicated in psychiatric diseases | Schizophrenia, mental illness | Neonatal hypotonia, cognitive and social deficits, psychotomimetic drug sensitivity | Late walking | [3, 20, 21] |
| *ZBED4* | 612552 | Regulatory protein, evolved from DNA transposon | Unknown | Neonatal hypotonia | Late walking | [3, 22] |
| *PIM3* | 610580 | Belongs to family of proto-oncogenes that encode threonine/serine protein kinases | Implicated in several cancers including pancreatic and Ewing sarcoma | Neonatal hypotonia | Reduced body size | [3, 23-25] |
| *MLC1* | 605908 | Transmembrane protein that associates with the N,K-ATPase beta-1 subunit (ATP1B1) in a multiprotein complex | Megaencephalic leukoencephalopathy with subcortical cysts | Neonatal hypotonia, delayed onset neurologic deterioration, cerebellar ataxia, spasticity, epilepsy, mild cognitive decline | Unknown | [3, 26, 27] |
| *PANX2* | 608421 | Unknown but when co-expressed with *PANX1* modifies voltage activated outward currents | Some association with focal cortical dysplasia | Neonatal hypotonia | Unknown | [3, 28, 29] |
| *SELO* | 607917 | Unknown but is a member of the seloproteins which contain the amino acid selenocysteine | Seloproteins implicated in many complex human diseases involving neurologic, bone, thyroid and immune functions | Seloproteinopathies associated with neonatal hypotonia, scoliosis, intellectual disability, epilepsy | Seloproteinsopathies associated with muscle weakness and atrophy, chrondrodysplasia, thyroid dysfunction | [3, 30, 31] |
| *TUBGCP6* | 610053 | Protein product a core component of centrosomes | Autosomal recessive microcephaly and chorioretinopathy | Neonatal hypotonia, microcephaly, cognitive deficits, epilepsy, arachnoid cysts | Short statue, retinopathy, cataracts, glaucoma, hyperopia, microopthalmia, polydactyly, triphalangeal thumbs | [3, 32, 33] |
| *MAPK11* | 602989 | Part of the mitogen-activated protein kinase family which are involved in transducing extracellular signals into cellular responses | Unknown | Neonatal hypotonia | Unknown | [3, 34] |
| *PLXNB2* | 604293 | Transmembrane receptor that is involved in axon guidance and cell migration in response to semaphorins | Unknown | Neonatal hypotonia, abnormal neural tube closure | Unknown | [3, 35, 36] |
| *PPP6R2* | 610877 | Protein phosphatase regulatory subnunit involved in substrate specificity, recruitment of substrates and localization of protein phosphatases | Unknown | Unknown | Unknown | [37] |
| *SBF1* | 603560 | Nuclear protein that prevents substrate dephosphorylation and is involved in epigenetic modification of signaling pathways important in growth and differentiation | Charcot-Marie-tooth disease, type 4B3 | Distal sensory impairment, areflexia, scoliosis, facial weakness, dysarthria | Progressive limb muscle weakness, pes planus, urinary incontinence, strabismus, opthalmoplegia, syndactyly | [38, 39] |
| *LMF2* | n/a | Unknown | Methylation of promoter region associated with amnestic mild cognitive impairment and Alzheimer’s disease | Unknown | Unknown | [40] |
| *NCAPH2* | 611230 | One of the non-structural maintenance of chromosomes subunits of condensing-II. The condensin complexes are involved in mitotic chromosome assembly and condensation | Unknown | Microcephaly, decreased brain weight | Unknown | [41, 42] |
| *SCO2* | 604272 | Metallochaperone involved in regulation of the cytochrome c oxidase complex in mitochondrial respiration | Myopia 6, fatal infantile cardioencephalomyopathy | Gliosis, hypotonia, encephalopathy, | Hypertrophic cardiomyopathy, lactic acidosis, myopia, abnormal breathing | [43-45] |
| *CHKB* | 612395 | Choline kinase that catalyzes the phosphorylation of choline by ATP in the presence of Mg(2+), yielding phosphocholine and ADP | Congenital megaconial muscular dystrophy | ID, hypotonia, delayed psychomotor development, microcephaly | Muscle wasting, cardiomyopathy, | [46, 47] |
| *MAPK8IP2* | 607755 | Expressed in neuronal and neuroendocrine cells, function poorly understood however needed for proper AMPA-type and NMDA-type evoked currents at cerebellar synapses | Unknown | Motor delay, reduced social interaction and impaired learning | Unknown | [48, 49] |
| *ARSA* | 607574 | Lysosomal enzyme arylsulfatase A | Metachromatic leukodystrophy | Mental deterioration, hypotonia, schizophrenia-like psychiatric symptoms | Rigidity, muscle weakness, unsteady gait | [50] |
| *SHANK3* | 606230 | Scaffolding protein enriched in post-synaptic densities of excitatory synapses | PMS, schizophrenia | Cognitive deficits, autistic symptoms, developmental delay, seizures, | Language abnormalities, facial dysmorphism | [51, 52] |

(ASD= autism spectrum disorder; ID= intellectual disability)

References

1. Online Mendelian Inheritance in Man, OMIM: McKusick-Nathans Institute of Genetic Medicine, Johns Hopkins University (Baltimore, MD); [cited 2018]. Available from: <https://omim.org/>.

2. Zhang L, Joshi AK, Smith S. Cloning, expression, characterization, and interaction of two components of a human mitochondrial fatty acid synthase. Malonyltransferase and acyl carrier protein. The Journal of biological chemistry. 2003;278(41):40067-74. Epub 2003/07/29. doi: 10.1074/jbc.M306121200. PubMed PMID: 12882974.

3. Sarasua SM, Dwivedi A, Boccuto L, Chen CF, Sharp JL, Rollins JD, et al. 22q13.2q13.32 genomic regions associated with severity of speech delay, developmental delay, and physical features in Phelan-McDermid syndrome. Genetics in medicine : official journal of the American College of Medical Genetics. 2014;16(4):318-28. Epub 2013/10/19. doi: 10.1038/gim.2013.144. PubMed PMID: 24136618.

4. Crittenden F, Thomas HR, Parant JM, Falany CN. Activity Suppression Behavior Phenotype in SULT4A1 Frameshift Mutant Zebrafish. Drug metabolism and disposition: the biological fate of chemicals. 2015;43(7):1037-44. Epub 2015/05/03. doi: 10.1124/dmd.115.064485. PubMed PMID: 25934576; PubMed Central PMCID: PMCPMC4468436.

5. Falany CN, Xie X, Wang J, Ferrer J, Falany JL. Molecular cloning and expression of novel sulphotransferase-like cDNAs from human and rat brain. The Biochemical journal. 2000;346 Pt 3:857-64. Epub 2000/03/04. PubMed PMID: 10698717; PubMed Central PMCID: PMCPMC1220923.

6. Tabet AC, Rolland T, Ducloy M, Levy J, Buratti J, Mathieu A, et al. A framework to identify contributing genes in patients with Phelan-McDermid syndrome. NPJ genomic medicine. 2017;2:32. Epub 2017/12/22. doi: 10.1038/s41525-017-0035-2. PubMed PMID: 29263841; PubMed Central PMCID: PMCPMC5677962.

7. Humphries AD, Streimann IC, Stojanovski D, Johnston AJ, Yano M, Hoogenraad NJ, et al. Dissection of the mitochondrial import and assembly pathway for human Tom40. The Journal of biological chemistry. 2005;280(12):11535-43. Epub 2005/01/13. doi: 10.1074/jbc.M413816200. PubMed PMID: 15644312.

8. Kitamoto T, Kitamoto A, Yoneda M, Hyogo H, Ochi H, Nakamura T, et al. Genome-wide scan revealed that polymorphisms in the PNPLA3, SAMM50, and PARVB genes are associated with development and progression of nonalcoholic fatty liver disease in Japan. Human genetics. 2013;132(7):783-92. Epub 2013/03/29. doi: 10.1007/s00439-013-1294-3. PubMed PMID: 23535911.

9. Rosenberger G, Jantke I, Gal A, Kutsche K. Interaction of alphaPIX (ARHGEF6) with beta-parvin (PARVB) suggests an involvement of alphaPIX in integrin-mediated signaling. Human molecular genetics. 2003;12(2):155-67. Epub 2002/12/25. PubMed PMID: 12499396.

10. Pei J, Grishin NV. Unexpected diversity in Shisa-like proteins suggests the importance of their roles as transmembrane adaptors. Cellular signalling. 2012;24(3):758-69. Epub 2011/11/29. doi: 10.1016/j.cellsig.2011.11.011. PubMed PMID: 22120523; PubMed Central PMCID: PMCPMC3295595.

11. De Smet L, De Beer P, Fryns JP. Cenani-Lenz syndrome in father and daughter. Genetic counseling (Geneva, Switzerland). 1996;7(2):153-7. Epub 1996/01/01. PubMed PMID: 8831136.

12. Argraves WS, Dickerson K, Burgess WH, Ruoslahti E. Fibulin, a novel protein that interacts with the fibronectin receptor beta subunit cytoplasmic domain. Cell. 1989;58(4):623-9. Epub 1989/08/25. PubMed PMID: 2527614.

13. Teive HA, Munhoz RP, Arruda WO, Raskin S, Werneck LC, Ashizawa T. Spinocerebellar ataxia type 10 - A review. Parkinsonism & related disorders. 2011;17(9):655-61. Epub 2011/05/03. doi: 10.1016/j.parkreldis.2011.04.001. PubMed PMID: 21531163.

14. Stenman JM, Rajagopal J, Carroll TJ, Ishibashi M, McMahon J, McMahon AP. Canonical Wnt signaling regulates organ-specific assembly and differentiation of CNS vasculature. Science (New York, NY). 2008;322(5905):1247-50. Epub 2008/11/22. doi: 10.1126/science.1164594. PubMed PMID: 19023080.

15. Rosso SB, Sussman D, Wynshaw-Boris A, Salinas PC. Wnt signaling through Dishevelled, Rac and JNK regulates dendritic development. Nature neuroscience. 2005;8(1):34-42. Epub 2004/12/21. doi: 10.1038/nn1374. PubMed PMID: 15608632.

16. Stanelle J, Tu-Rapp H, Putzer BM. A novel mitochondrial protein DIP mediates E2F1-induced apoptosis independently of p53. Cell death and differentiation. 2005;12(4):347-57. Epub 2004/11/27. doi: 10.1038/sj.cdd.4401532. PubMed PMID: 15565177.

17. Kim TJ, Mitsutake S, Igarashi Y. The interaction between the pleckstrin homology domain of ceramide kinase and phosphatidylinositol 4,5-bisphosphate regulates the plasma membrane targeting and ceramide 1-phosphate levels. Biochemical and biophysical research communications. 2006;342(2):611-7. Epub 2006/02/21. doi: 10.1016/j.bbrc.2006.01.170. PubMed PMID: 16488390.

18. Tom Tang Y, Emtage P, Funk WD, Hu T, Arterburn M, Park EE, et al. TAFA: a novel secreted family with conserved cysteine residues and restricted expression in the brain. Genomics. 2004;83(4):727-34. Epub 2004/03/19. doi: 10.1016/j.ygeno.2003.10.006. PubMed PMID: 15028294.

19. De Strooper LM, Meijer CJ, Berkhof J, Hesselink AT, Snijders PJ, Steenbergen RD, et al. Methylation analysis of the FAM19A4 gene in cervical scrapes is highly efficient in detecting cervical carcinomas and advanced CIN2/3 lesions. Cancer prevention research (Philadelphia, Pa). 2014;7(12):1251-7. Epub 2014/10/05. doi: 10.1158/1940-6207.capr-14-0237. PubMed PMID: 25281488.

20. Fryland T, Christensen JH, Pallesen J, Mattheisen M, Palmfeldt J, Bak M, et al. Identification of the BRD1 interaction network and its impact on mental disorder risk. Genome medicine. 2016;8(1):53. Epub 2016/05/05. doi: 10.1186/s13073-016-0308-x. PubMed PMID: 27142060; PubMed Central PMCID: PMCPMC4855718.

21. Qvist P, Eskildsen SF, Hansen B, Baragji M, Ringgaard S, Roovers J, et al. Brain volumetric alterations accompanied with loss of striatal medium-sized spiny neurons and cortical parvalbumin expressing interneurons in Brd1(+/-) mice. Scientific reports. 2018;8(1):16486. Epub 2018/11/09. doi: 10.1038/s41598-018-34729-5. PubMed PMID: 30405140; PubMed Central PMCID: PMCPMC6220279.

22. Hayward A, Ghazal A, Andersson G, Andersson L, Jern P. ZBED evolution: repeated utilization of DNA transposons as regulators of diverse host functions. PloS one. 2013;8(3):e59940. Epub 2013/03/28. doi: 10.1371/journal.pone.0059940. PubMed PMID: 23533661; PubMed Central PMCID: PMCPMC3606216.

23. Mikkers H, Nawijn M, Allen J, Brouwers C, Verhoeven E, Jonkers J, et al. Mice deficient for all PIM kinases display reduced body size and impaired responses to hematopoietic growth factors. Molecular and cellular biology. 2004;24(13):6104-15. Epub 2004/06/17. doi: 10.1128/mcb.24.13.6104-6115.2004. PubMed PMID: 15199164; PubMed Central PMCID: PMCPMC480904.

24. Li YY, Popivanova BK, Nagai Y, Ishikura H, Fujii C, Mukaida N. Pim-3, a proto-oncogene with serine/threonine kinase activity, is aberrantly expressed in human pancreatic cancer and phosphorylates bad to block bad-mediated apoptosis in human pancreatic cancer cell lines. Cancer research. 2006;66(13):6741-7. Epub 2006/07/05. doi: 10.1158/0008-5472.can-05-4272. PubMed PMID: 16818649.

25. Deneen B, Welford SM, Ho T, Hernandez F, Kurland I, Denny CT. PIM3 proto-oncogene kinase is a common transcriptional target of divergent EWS/ETS oncoproteins. Molecular and cellular biology. 2003;23(11):3897-908. Epub 2003/05/16. PubMed PMID: 12748291; PubMed Central PMCID: PMCPMC155223.

26. Lanciotti A, Brignone MS, Molinari P, Visentin S, De Nuccio C, Macchia G, et al. Megalencephalic leukoencephalopathy with subcortical cysts protein 1 functionally cooperates with the TRPV4 cation channel to activate the response of astrocytes to osmotic stress: dysregulation by pathological mutations. Human molecular genetics. 2012;21(10):2166-80. Epub 2012/02/14. doi: 10.1093/hmg/dds032. PubMed PMID: 22328087.

27. Lopez-Hernandez T, Ridder MC, Montolio M, Capdevila-Nortes X, Polder E, Sirisi S, et al. Mutant GlialCAM causes megalencephalic leukoencephalopathy with subcortical cysts, benign familial macrocephaly, and macrocephaly with retardation and autism. American journal of human genetics. 2011;88(4):422-32. Epub 2011/03/23. doi: 10.1016/j.ajhg.2011.02.009. PubMed PMID: 21419380; PubMed Central PMCID: PMCPMC3071909.

28. Bruzzone R, Hormuzdi SG, Barbe MT, Herb A, Monyer H. Pannexins, a family of gap junction proteins expressed in brain. Proceedings of the National Academy of Sciences of the United States of America. 2003;100(23):13644-9. Epub 2003/11/05. doi: 10.1073/pnas.2233464100. PubMed PMID: 14597722; PubMed Central PMCID: PMCPMC263867.

29. Li S, Zang Z, He J, Chen X, Yu S, Pei Y, et al. Expression of pannexin 1 and 2 in cortical lesions from intractable epilepsy patients with focal cortical dysplasia. Oncotarget. 2017;8(4):6883-95. Epub 2016/12/31. doi: 10.18632/oncotarget.14317. PubMed PMID: 28036289; PubMed Central PMCID: PMCPMC5351677.

30. Kryukov GV, Kryukov VM, Gladyshev VN. New mammalian selenocysteine-containing proteins identified with an algorithm that searches for selenocysteine insertion sequence elements. The Journal of biological chemistry. 1999;274(48):33888-97. Epub 1999/11/24. PubMed PMID: 10567350.

31. Schweizer U, Fradejas-Villar N. Why 21? The significance of selenoproteins for human health revealed by inborn errors of metabolism. FASEB journal : official publication of the Federation of American Societies for Experimental Biology. 2016;30(11):3669-81. Epub 2016/11/03. doi: 10.1096/fj.201600424. PubMed PMID: 27473727.

32. Martin CA, Ahmad I, Klingseisen A, Hussain MS, Bicknell LS, Leitch A, et al. Mutations in PLK4, encoding a master regulator of centriole biogenesis, cause microcephaly, growth failure and retinopathy. Nature genetics. 2014;46(12):1283-92. Epub 2014/10/27. doi: 10.1038/ng.3122. PubMed PMID: 25344692; PubMed Central PMCID: PMCPMC4676084.

33. Murphy SM, Preble AM, Patel UK, O'Connell KL, Dias DP, Moritz M, et al. GCP5 and GCP6: two new members of the human gamma-tubulin complex. Molecular biology of the cell. 2001;12(11):3340-52. Epub 2001/11/06. doi: 10.1091/mbc.12.11.3340. PubMed PMID: 11694571; PubMed Central PMCID: PMCPMC60259.

34. Jiang Y, Chen C, Li Z, Guo W, Gegner JA, Lin S, et al. Characterization of the structure and function of a new mitogen-activated protein kinase (p38beta). The Journal of biological chemistry. 1996;271(30):17920-6. Epub 1996/07/26. PubMed PMID: 8663524.

35. Perrot V, Vazquez-Prado J, Gutkind JS. Plexin B regulates Rho through the guanine nucleotide exchange factors leukemia-associated Rho GEF (LARG) and PDZ-RhoGEF. The Journal of biological chemistry. 2002;277(45):43115-20. Epub 2002/08/17. doi: 10.1074/jbc.M206005200. PubMed PMID: 12183458.

36. Friedel RH, Kerjan G, Rayburn H, Schuller U, Sotelo C, Tessier-Lavigne M, et al. Plexin-B2 controls the development of cerebellar granule cells. The Journal of neuroscience : the official journal of the Society for Neuroscience. 2007;27(14):3921-32. Epub 2007/04/06. doi: 10.1523/jneurosci.4710-06.2007. PubMed PMID: 17409257.

37. Stefansson B, Brautigan DL. Protein phosphatase 6 subunit with conserved Sit4-associated protein domain targets IkappaBepsilon. The Journal of biological chemistry. 2006;281(32):22624-34. Epub 2006/06/14. doi: 10.1074/jbc.M601772200. PubMed PMID: 16769727.

38. Cui X, De Vivo I, Slany R, Miyamoto A, Firestein R, Cleary ML. Association of SET domain and myotubularin-related proteins modulates growth control. Nature genetics. 1998;18(4):331-7. Epub 1998/04/16. doi: 10.1038/ng0498-331. PubMed PMID: 9537414.

39. Bohlega S, Alazami AM, Cupler E, Al-Hindi H, Ibrahim E, Alkuraya FS. A novel syndromic form of sensory-motor polyneuropathy is linked to chromosome 22q13.31-q13.33. Clinical genetics. 2011;79(2):193-5. Epub 2011/01/08. doi: 10.1111/j.1399-0004.2010.01524.x. PubMed PMID: 21210780.

40. Shinagawa S, Kobayashi N, Nagata T, Kusaka A, Yamada H, Kondo K, et al. DNA methylation in the NCAPH2/LMF2 promoter region is associated with hippocampal atrophy in Alzheimer's disease and amnesic mild cognitive impairment patients. Neuroscience letters. 2016;629:33-7. Epub 2016/06/30. doi: 10.1016/j.neulet.2016.06.055. PubMed PMID: 27356276.

41. Ono T, Losada A, Hirano M, Myers MP, Neuwald AF, Hirano T. Differential contributions of condensin I and condensin II to mitotic chromosome architecture in vertebrate cells. Cell. 2003;115(1):109-21. Epub 2003/10/09. PubMed PMID: 14532007.

42. Martin CA, Murray JE, Carroll P, Leitch A, Mackenzie KJ, Halachev M, et al. Mutations in genes encoding condensin complex proteins cause microcephaly through decatenation failure at mitosis. Genes & development. 2016;30(19):2158-72. Epub 2016/11/01. doi: 10.1101/gad.286351.116. PubMed PMID: 27737959; PubMed Central PMCID: PMCPMC5088565.

43. Leary SC, Kaufman BA, Pellecchia G, Guercin GH, Mattman A, Jaksch M, et al. Human SCO1 and SCO2 have independent, cooperative functions in copper delivery to cytochrome c oxidase. Human molecular genetics. 2004;13(17):1839-48. Epub 2004/07/02. doi: 10.1093/hmg/ddh197. PubMed PMID: 15229189.

44. Papadopoulou LC, Sue CM, Davidson MM, Tanji K, Nishino I, Sadlock JE, et al. Fatal infantile cardioencephalomyopathy with COX deficiency and mutations in SCO2, a COX assembly gene. Nature genetics. 1999;23(3):333-7. Epub 1999/11/05. doi: 10.1038/15513. PubMed PMID: 10545952.

45. Tran-Viet KN, Powell C, Barathi VA, Klemm T, Maurer-Stroh S, Limviphuvadh V, et al. Mutations in SCO2 are associated with autosomal-dominant high-grade myopia. American journal of human genetics. 2013;92(5):820-6. Epub 2013/05/07. doi: 10.1016/j.ajhg.2013.04.005. PubMed PMID: 23643385; PubMed Central PMCID: PMCPMC3644634.

46. Ishidate K. Choline/ethanolamine kinase from mammalian tissues. Biochimica et biophysica acta. 1997;1348(1-2):70-8. Epub 1997/11/25. PubMed PMID: 9370318.

47. Mitsuhashi S, Ohkuma A, Talim B, Karahashi M, Koumura T, Aoyama C, et al. A congenital muscular dystrophy with mitochondrial structural abnormalities caused by defective de novo phosphatidylcholine biosynthesis. American journal of human genetics. 2011;88(6):845-51. Epub 2011/06/15. doi: 10.1016/j.ajhg.2011.05.010. PubMed PMID: 21665002; PubMed Central PMCID: PMCPMC3113344.

48. Giza J, Urbanski MJ, Prestori F, Bandyopadhyay B, Yam A, Friedrich V, et al. Behavioral and cerebellar transmission deficits in mice lacking the autism-linked gene islet brain-2. The Journal of neuroscience : the official journal of the Society for Neuroscience. 2010;30(44):14805-16. Epub 2010/11/05. doi: 10.1523/jneurosci.1161-10.2010. PubMed PMID: 21048139; PubMed Central PMCID: PMCPMC3200367.

49. Negri S, Oberson A, Steinmann M, Sauser C, Nicod P, Waeber G, et al. cDNA cloning and mapping of a novel islet-brain/JNK-interacting protein. Genomics. 2000;64(3):324-30. Epub 2000/04/11. doi: 10.1006/geno.2000.6129. PubMed PMID: 10756100.

50. Greenfield JG. A Form of Progressive Cerebral Sclerosis in Infants associated with Primary Degeneration of the Interfascicular Glia. Proceedings of the Royal Society of Medicine. 1933;26(6):690-7. Epub 1933/04/01. PubMed PMID: 19989245; PubMed Central PMCID: PMCPMC2204463.

51. Yi F, Danko T, Botelho SC, Patzke C, Pak C, Wernig M, et al. Autism-associated SHANK3 haploinsufficiency causes Ih channelopathy in human neurons. Science (New York, NY). 2016;352(6286):aaf2669. Epub 2016/03/12. doi: 10.1126/science.aaf2669. PubMed PMID: 26966193; PubMed Central PMCID: PMCPMC4901875.

52. Bonaglia MC, Giorda R, Borgatti R, Felisari G, Gagliardi C, Selicorni A, et al. Disruption of the ProSAP2 gene in a t(12;22)(q24.1;q13.3) is associated with the 22q13.3 deletion syndrome. American journal of human genetics. 2001;69(2):261-8. Epub 2001/06/30. doi: 10.1086/321293. PubMed PMID: 11431708; PubMed Central PMCID: PMCPMC1235301.
