# Supplementary material for: Functional genomics analysis of Phelan-McDermid syndrome 22q13 region during human neurodevelopment
Source: PLoS One. 2019 Mar 15;14(3):e0213921. doi: 10.1371/journal.pone.0213921 (PMC6420160; doi:10.1371/journal.pone.0213921)
Supplement: S2 Table — Developmental time period when RPKM >5 also shown. (1: 16 pcw– 17 pcw, 2: 19 pcw– 24 pcw, 3: 4 mos– 1 yr, 4: 2 yrs– 4 yrs, 5:8 yrs– 13 yrs, 6: 15 yrs– 21 yrs, 7: 23 yrs– 40 yrs). (pcw = post-conception weeks, mos = months, yrs = years). (DOCX) [file pone.0213921.s002.docx]

**S2 Table.** Gene function and clinical phenotypes associated with the 18 protein-coding genes with more than one read > 5 RPKM but with overall average gene expression <5. Developmental time period when RPKM >5 also shown. (1: 16 pcw – 17 pcw, 2: 19 pcw – 24 pcw, 3: 4 mos – 1 yr, 4: 2 yrs – 4 yrs, 5:8 yrs – 13 yrs, 6: 15 yrs – 21 yrs, 7: 23 yrs – 40 yrs). (pcw = post-conception weeks, mos = months, yrs = years).

| **gene symbol** | **OMIM number [1]** | **Developmental time period when RPKM >5** | **Function of protein product** | **associated disease** | **Associated neurologic phenotype** | **Other associated phenotype** | **Ref** |
| --- | --- | --- | --- | --- | --- | --- | --- |
| PNPLA3 | 609567 | 1 | Unknown | Variants associated with non-alcoholic fatty liver disease and alcoholic cirrhosis | speech abnormalities, absence of ASD | Hair pulling, male genital anomalies | [2-4] |
| PRR5 | 609406 | 2,3 | Component of the mammalian target of rapamycin 2 (mTOR2 complex) | Unknown | Unknown | Unknown | [5] |
| PHF21B | 616727 | 1,2 | Nuclear protein that functions to reduce cell migration and colony formation when overexpressed | Implicated as tumor suppressor gene in head and neck cancers | speech abnormalities, absence of ASD, facial asymmetry, abnormal reflexes | Hair pulling, male genital anomalies, dysplastic toenails, large hands | [2, 6] |
| NUP50 | 604646 | 1-3, 5-6 | Nucleoprotein that is part of the nuclear pore complex that allows bidirectional flow of macromolecules through nuclear envelope | Unknown | speech abnormalities, absence of ASD, facial asymmetry, abnormal reflexes, neonatal hypotonia | Hair pulling, male genital anomalies, dysplastic toenails, large hands, tall stature | [2, 7] |
| FAM118A | n/a | 2, 4 | Unknown | Unknown | Neonatal hypotonia, speech abnormalities, absence of ASD, facial asymmetry, abnormal reflexes | Dysplastic toenails, large hands, tall stature, hair pulling, male genital anomalies | [2] |
| PPARA | 170998 | 2,4 | Nuclear transcription factor that regulates expression of fatty acid oxidation enzymes | Increased susceptibility to hyperapobetalipoproteinemia | Neonatal hypotonia, speech abnormalities, absence of ASD, macrocephaly | Dysplastic toenails, large hands, late to walk, hair pulling, male genital anomalies | [2, 8] |
| TTC38 | n/a | 2,4 | Unknown | Unknown | Neonatal hypotonia, speech abnormalities, absence of ASD, macrocephaly | Dysplastic toenails, large hands, late to walk, hair pulling, male genital anomalies | [2] |
| GTSE1 | 607477 | 1 | Protein localized to microtubules and functions in cell cycle | Unknown | Neonatal hypotonia, speech abnormalities, absence of ASD, macrocephaly | Dysplastic toenails, large hands, late to walk, hair pulling, male genital anomalies | [2, 9] |
| CELSR1 | 604523 | 1 | Helps establish polarity of hair follicles in anterio-posterior axis | Increased susceptibility to neural tube defects | Neonatal hypotonia, speech abnormalities, absence of ASD, macrocephaly | Dysplastic toenails, large hands, late to walk, hair pulling, male genital anomalies | [2, 10-12] |
| TBC1D22A | 616879 | 2, 5-7 | Golgi protein predicted to function as GTPase- activating protein for RAB33 | Unknown | Neonatal hypotonia, absence of ASD, macrocephaly | Dysplastic toenails, large hands, late to walk, hair pulling | [2, 13] |
| ALG12 | 607144 | 1-6 | Catalyzes the addition of the 8^th^ mannose residue onto the lipid-linked oligosaccharide precursor during the synthesis of complex oligosaccharide-linked glycoproteins within the Golgi apparatus and endoplasmic reticulum | Congenital disorder of glycosylation, type Ig | Neonatal hypotonia, microcephaly | Late to walk, anorexia, psychomotor retardation, facial dysmorphism, failure to thrive, blindness, deafness, male genital hypoplasia, cardiac abnormalities, generalized edema | [2, 14, 15] |
| CRELD2 | 607171 | 1-4 | Transmembrane protein with epidermal-like growth factor repeats that mediate protein-protein interactions | Unknown | Neonatal hypotonia | Late to walk | [2, 16] |
| TRABD | n/a | 1-4, 6-7 | Unknown | Unknown | Neonatal hypotonia | Unknown | [2] |
| HDAC10 | 608544 | 1-4, 7 | Histone deacetylase | Unknown | Neonatal hypotonia | Unknown | [2, 17] |
| MAPK12 | 602399 | 1-7 | Part of mitogen-activated protein kinase family of proteins that mediate extracellular signaling | Unknown | Neonatal hypotonia | Unknown | [2, 18] |
| TYMP | 131222 | 2 | Catalyzes the phosphorylation of thymidine to thymine, promotes neuronal survival, chemotactic for endothelial cells acting as an angiogenic factor | Mitochondrial DNA depletion syndrome 1 | Polyneuropathy, leukoencephalopathy | Muscle atrophy, gastrointestinal dysmotility, external opthalmoplegia, ptosis | [19, 20] |
| CPT1B | 601987 | 1-7 | Mitochondrial carnitine palmitoyltransferase which allows the transport of long-chain fatty acyl-CoA’s from cytoplasm to the mitochonidrial membrane for beta-oxidation | Unknown | Unknown | Cold intolerance | [21] |
| RABL2B | 605413 | 1-2 | Member of RAB family of proteins, which are a group a GTP-binding proteins involved in exocytic and endocytic pathways. Also has role in intraflagellar transport and ciliogenesis | Unknown | Unknown | Infertility, polydactyly, retinal degeneration | [22, 23] |

(ref = references; ASD= autism spectrum disorder; ID= intellectual disability)

References

1. Online Mendelian Inheritance in Man, OMIM: McKusick-Nathans Institute of Genetic Medicine, Johns Hopkins University (Baltimore, MD); [cited 2018]. Available from: <https://omim.org/>.

2. Sarasua SM, Dwivedi A, Boccuto L, Chen CF, Sharp JL, Rollins JD, et al. 22q13.2q13.32 genomic regions associated with severity of speech delay, developmental delay, and physical features in Phelan-McDermid syndrome. Genetics in medicine : official journal of the American College of Medical Genetics. 2014;16(4):318-28. Epub 2013/10/19. doi: 10.1038/gim.2013.144. PubMed PMID: 24136618.

3. Romeo S, Kozlitina J, Xing C, Pertsemlidis A, Cox D, Pennacchio LA, et al. Genetic variation in PNPLA3 confers susceptibility to nonalcoholic fatty liver disease. Nature genetics. 2008;40(12):1461-5. Epub 2008/09/30. doi: 10.1038/ng.257. PubMed PMID: 18820647; PubMed Central PMCID: PMCPMC2597056.

4. Tian C, Stokowski RP, Kershenobich D, Ballinger DG, Hinds DA. Variant in PNPLA3 is associated with alcoholic liver disease. Nature genetics. 2010;42(1):21-3. Epub 2009/12/01. doi: 10.1038/ng.488. PubMed PMID: 19946271.

5. Pearce LR, Huang X, Boudeau J, Pawlowski R, Wullschleger S, Deak M, et al. Identification of Protor as a novel Rictor-binding component of mTOR complex-2. The Biochemical journal. 2007;405(3):513-22. Epub 2007/04/28. doi: 10.1042/bj20070540. PubMed PMID: 17461779; PubMed Central PMCID: PMCPMC2267312.

6. Bertonha FB, Barros Filho Mde C, Kuasne H, Dos Reis PP, da Costa Prando E, Munoz JJ, et al. PHF21B as a candidate tumor suppressor gene in head and neck squamous cell carcinomas. Molecular oncology. 2015;9(2):450-62. Epub 2014/12/03. doi: 10.1016/j.molonc.2014.09.009. PubMed PMID: 25454821; PubMed Central PMCID: PMCPMC5528662.

7. Lindsay ME, Plafker K, Smith AE, Clurman BE, Macara IG. Npap60/Nup50 is a tri-stable switch that stimulates importin-alpha:beta-mediated nuclear protein import. Cell. 2002;110(3):349-60. Epub 2002/08/15. PubMed PMID: 12176322.

8. Djouadi F, Weinheimer CJ, Saffitz JE, Pitchford C, Bastin J, Gonzalez FJ, et al. A gender-related defect in lipid metabolism and glucose homeostasis in peroxisome proliferator- activated receptor alpha- deficient mice. The Journal of clinical investigation. 1998;102(6):1083-91. Epub 1998/09/17. doi: 10.1172/jci3949. PubMed PMID: 9739042; PubMed Central PMCID: PMCPMC509091.

9. Monte M, Collavin L, Lazarevic D, Utrera R, Dragani TA, Schneider C. Cloning, chromosome mapping and functional characterization of a human homologue of murine gtse-1 (B99) gene. Gene. 2000;254(1-2):229-36. Epub 2000/09/07. PubMed PMID: 10974554.

10. Devenport D, Fuchs E. Planar polarization in embryonic epidermis orchestrates global asymmetric morphogenesis of hair follicles. Nature cell biology. 2008;10(11):1257-68. Epub 2008/10/14. doi: 10.1038/ncb1784. PubMed PMID: 18849982; PubMed Central PMCID: PMCPMC2607065.

11. Allache R, De Marco P, Merello E, Capra V, Kibar Z. Role of the planar cell polarity gene CELSR1 in neural tube defects and caudal agenesis. Birth defects research Part A, Clinical and molecular teratology. 2012;94(3):176-81. Epub 2012/03/01. doi: 10.1002/bdra.23002. PubMed PMID: 22371354.

12. Robinson A, Escuin S, Doudney K, Vekemans M, Stevenson RE, Greene ND, et al. Mutations in the planar cell polarity genes CELSR1 and SCRIB are associated with the severe neural tube defect craniorachischisis. Human mutation. 2012;33(2):440-7. Epub 2011/11/19. doi: 10.1002/humu.21662. PubMed PMID: 22095531; PubMed Central PMCID: PMCPMC4772123.

13. Greninger AL, Knudsen GM, Betegon M, Burlingame AL, DeRisi JL. ACBD3 interaction with TBC1 domain 22 protein is differentially affected by enteroviral and kobuviral 3A protein binding. mBio. 2013;4(2):e00098-13. Epub 2013/04/11. doi: 10.1128/mBio.00098-13. PubMed PMID: 23572552; PubMed Central PMCID: PMCPMC3622926.

14. Kranz C, Basinger AA, Gucsavas-Calikoglu M, Sun L, Powell CM, Henderson FW, et al. Expanding spectrum of congenital disorder of glycosylation Ig (CDG-Ig): sibs with a unique skeletal dysplasia, hypogammaglobulinemia, cardiomyopathy, genital malformations, and early lethality. American journal of medical genetics Part A. 2007;143a(12):1371-8. Epub 2007/05/17. doi: 10.1002/ajmg.a.31791. PubMed PMID: 17506107.

15. Chantret I, Dupre T, Delenda C, Bucher S, Dancourt J, Barnier A, et al. Congenital disorders of glycosylation type Ig is defined by a deficiency in dolichyl-P-mannose:Man7GlcNAc2-PP-dolichyl mannosyltransferase. The Journal of biological chemistry. 2002;277(28):25815-22. Epub 2002/05/02. doi: 10.1074/jbc.M203285200. PubMed PMID: 11983712.

16. Rupp PA, Fouad GT, Egelston CA, Reifsteck CA, Olson SB, Knosp WM, et al. Identification, genomic organization and mRNA expression of CRELD1, the founding member of a unique family of matricellular proteins. Gene. 2002;293(1-2):47-57. Epub 2002/07/26. PubMed PMID: 12137942.

17. Kao HY, Lee CH, Komarov A, Han CC, Evans RM. Isolation and characterization of mammalian HDAC10, a novel histone deacetylase. The Journal of biological chemistry. 2002;277(1):187-93. Epub 2001/10/26. doi: 10.1074/jbc.M108931200. PubMed PMID: 11677242.

18. Goedert M, Hasegawa J, Craxton M, Leversha MA, Clegg S. Assignment of the human stress-activated protein kinase-3 gene (SAPK3) to chromosome 22q13.3 by fluorescence in situ hybridization. Genomics. 1997;41(3):501-2. Epub 1997/05/01. doi: 10.1006/geno.1997.4633. PubMed PMID: 9169156.

19. Taanman JW, Daras M, Albrecht J, Davie CA, Mallam EA, Muddle JR, et al. Characterization of a novel TYMP splice site mutation associated with mitochondrial neurogastrointestinal encephalomyopathy (MNGIE). Neuromuscular disorders : NMD. 2009;19(2):151-4. Epub 2008/12/06. doi: 10.1016/j.nmd.2008.11.002. PubMed PMID: 19056268.

20. Griffiths L, Stratford IJ. Platelet-derived endothelial cell growth factor thymidine phosphorylase in tumour growth and response to therapy. British journal of cancer. 1997;76(6):689-93. Epub 1997/01/01. PubMed PMID: 9310231; PubMed Central PMCID: PMCPMC2228052.

21. Ji S, You Y, Kerner J, Hoppel CL, Schoeb TR, Chick WS, et al. Homozygous carnitine palmitoyltransferase 1b (muscle isoform) deficiency is lethal in the mouse. Molecular genetics and metabolism. 2008;93(3):314-22. Epub 2007/11/21. doi: 10.1016/j.ymgme.2007.10.006. PubMed PMID: 18023382; PubMed Central PMCID: PMCPMC2270477.

22. Nishijima Y, Hagiya Y, Kubo T, Takei R, Katoh Y, Nakayama K. RABL2 interacts with the intraflagellar transport-B complex and CEP19 and participates in ciliary assembly. Molecular biology of the cell. 2017;28(12):1652-66. Epub 2017/04/22. doi: 10.1091/mbc.E17-01-0017. PubMed PMID: 28428259; PubMed Central PMCID: PMCPMC5469608.

23. Kanie T, Abbott KL, Mooney NA, Plowey ED, Demeter J, Jackson PK. The CEP19-RABL2 GTPase Complex Binds IFT-B to Initiate Intraflagellar Transport at the Ciliary Base. Developmental cell. 2017;42(1):22-36.e12. Epub 2017/06/20. doi: 10.1016/j.devcel.2017.05.016. PubMed PMID: 28625565; PubMed Central PMCID: PMCPMC5556974.
