# Supplementary material for: Functional genomics analysis of Phelan-McDermid syndrome 22q13 region during human neurodevelopment
Source: PLoS One. 2019 Mar 15;14(3):e0213921. doi: 10.1371/journal.pone.0213921 (PMC6420160; doi:10.1371/journal.pone.0213921)
Supplement: S3 Table — The average expression in RPKM and SD of each of the 65 protein coding genes within the PMS region is shown. Genes are ordered by most proximal to most distal on chromosome. (DOCX) [file pone.0213921.s003.docx]

**S3 Table.** Expression of genes within 22q13 region. The average expression in RPKM and SD of each of the 65 protein coding genes within the PMS region is shown. Genes are ordered by most proximal to most distal on chromosome.

| gene symbol | ENSEMBL gene ID | gene ID | entrez ID | average expression (RPKM) | SD | genomic coordinates from UCSC genome browser [1] |
| --- | --- | --- | --- | --- | --- | --- |
| MCAT | ENSG00000100294 | 27094 | 27349 | 11.18002459 | 3.920677468 | chr22:43,132,205-43,143,396 |
| SULT4A1 | ENSG00000130540 | 25579 | 25830 | 61.81100664 | 50.1464457 | chr22:43,824,506-43,862,497 |
| PNPLA5 | ENSG00000100341 | 80885 | 150379 | 0.241031857 | 0.316538763 | chr22:43,879,677-43,892,012 |
| PNPLA3 | ENSG00000100344 | 56140 | 80339 | 2.723800844 | 2.048566089 | chr22:43,923,738-43,947,567 |
| SAMM50 | ENSG00000100347 | 25562 | 25813 | 7.59079345 | 2.005860795 | chr22:43,955,380-43,996,531 |
| PARVB | ENSG00000188677 | 29515 | 29780 | 5.09439891 | 2.33524518 | chr22:43,999,163-44,169,232 |
| PARVG | ENSG00000138964 | 40731 | 64098 | 0.247582412 | 0.228970247 | chr22:44,170,227-44,208,468 |
| KIAA1644 | ENSG00000138944 | 59523 | 85352 | 18.49722783 | 14.91144098 | chr22:44,249,685-44,300,945 |
| LDOC1L | ENSG00000188636 | 58457 | 84247 | 32.60779911 | 19.18594219 | chr22:44,492,572-44,498,298 |
| PRR5 | ENSG00000186654 | 34904 | 55615 | 3.843271258 | 2.611406588 | chr22:44,702,233-44,862,671 |
| ARHGAP8 | ENSG00000241484 | 142313 | 553158 | 1.440109389 | 0.793725528 | chr22:44,752,606-44,862,784 |
| PHF21B | ENSG00000056487 | 76622 | 112885 | 3.603968962 | 5.078056229 | chr22:44,881,162-45,009,700 |
| NUP50 | ENSG00000093000 | 10604 | 10762 | 4.645198609 | 1.90183837 | chr22:45,163,841-45,188,015 |
| KIAA0930 | ENSG00000100364 | 23068 | 23313 | 20.87300748 | 7.943523692 | chr22:45,190,338-45,240,769 |
| UPK3A | ENSG00000100373 | 7337 | 7380 | 0.061845408 | 0.094754132 | chr22:45,284,982-45,295,874 |
| FAM118A | ENSG00000100376 | 34296 | 55007 | 1.834052948 | 0.936705973 | chr22:45,308,982-45,341,955 |
| SMC1B | ENSG00000077935 | 26872 | 27127 | 0.026002519 | 0.029841086 | chr22:45,344,063-45,413,567 |
| RIBC2 | ENSG00000128408 | 25899 | 26150 | 0.285182153 | 0.549142771 | chr22:45,413,691-45,432,496 |
| FBLN1 | ENSG00000077942 | 2177 | 2192 | 11.44024716 | 11.3889085 | chr22:45,502,891-45,601,135 |
| ATXN10 | ENSG00000130638 | 25563 | 25814 | 48.24386939 | 17.30643916 | chr22:45,671,799-45,845,307 |
| WNT7B | ENSG00000188064 | 7434 | 7477 | 8.435205828 | 12.88070661 | chr22:45,920,362-45,977,129 |
| PPARA | ENSG00000186951 | 5433 | 5465 | 1.291512508 | 1.106402349 | chr22:46,150,596-46,243,756 |
| PKDREJ | ENSG00000130943 | 10196 | 10343 | 0.062685302 | 0.048252724 | chr22:46,255,663-46,263,355 |
| TTC38 | ENSG00000075234 | 34309 | 55020 | 2.640272964 | 1.750978745 | chr22:46,267,964-46,294,008 |
| GTSE1 | ENSG00000075218 | 30992 | 51512 | 1.464987933 | 3.651474316 | chr22:46,296,741-46,330,810 |
| TRMU | ENSG00000100416 | 34976 | 55687 | 1.990206679 | 0.751032398 | chr22:46,335,425-46,357,340 |
| CELSR1 | ENSG00000075275 | 9476 | 9620 | 1.363488779 | 3.66657061 | chr22:46,360,834-46,537,170 |
| GRAMD4 | ENSG00000075240 | 22906 | 23151 | 8.639958973 | 3.477407346 | chr22:46,620,402-46,679,785 |
| CERK | ENSG00000100422 | 41354 | 64781 | 17.89005765 | 9.682614398 | chr22:46,684,411-46,738,261 |
| TBC1D22A | ENSG00000054611 | 25522 | 25771 | 3.36219829 | 0.993004558 | chr22:46,762,621-47,175,693 |
| FAM19A5 | ENSG00000219438 | 25566 | 25817 | 24.07256069 | 9.163179514 | chr22:48,576,306-48,751,932 |
| BRD1 | ENSG00000100425 | 23527 | 23774 | 6.933873723 | 4.754964032 | chr22:49,773,283-49,825,900 |
| ZBED4 | ENSG00000100426 | 9745 | 9889 | 6.416260813 | 6.21331736 | chr22:49,853,842-49,890,078 |
| ALG12 | ENSG00000182858 | 54926 | 79087 | 4.47194541 | 1.375814778 | chr22:49,900,229-49,918,458 |
| CRELD2 | ENSG00000184164 | 55013 | 79174 | 3.551302826 | 1.168177573 | chr22:49,918,696-49,927,528 |
| PIM3 | ENSG00000198355 | 128954 | 415116 | 10.20112478 | 3.569706354 | chr22:49,960,513-49,964,080 |
| IL17REL | ENSG00000188263 | 125352 | 400935 | 0.073777132 | 0.134876009 | chr22:49,994,513-50,012,659 |
| MLC1 | ENSG00000100427 | 22964 | 23209 | 45.39486705 | 56.38946547 | chr22:50,059,391-50,085,902 |
| MOV10L1 | ENSG00000073146 | 33747 | 54456 | 0.235004758 | 0.166904514 | chr22:50,089,879-50,161,618 |
| PANX2 | ENSG00000073150 | 35945 | 56666 | 11.12746229 | 9.11062093 | chr22:50,170,731-50,180,294 |
| TRABD | ENSG00000170638 | 56106 | 80305 | 4.435766733 | 2.42233783 | chr22:50,185,917-50,199,598 |
| SELO | ENSG00000073169 | 57884 | 83642 | 6.792388817 | 4.547407345 | chr22:50,200,979-50,217,616 |
| TUBGCP6 | ENSG00000128159 | 59548 | 85378 | 6.133276328 | 3.243165899 | chr22:50,217,689-50,244,564 |
| HDAC10 | ENSG00000100429 | 58150 | 83933 | 3.576573548 | 2.120956084 | chr22:50,245,183-50,251,385 |
| MAPK12 | ENSG00000188130 | 6266 | 6300 | 3.691156532 | 1.834908022 | chr22:50,252,903-50,261,825 |
| MAPK11 | ENSG00000185386 | 5568 | 5600 | 13.14177988 | 5.994701641 | chr22:50,263,713-50,270,393 |
| PLXNB2 | ENSG00000196576 | 23407 | 23654 | 15.20185746 | 13.15239782 | chr22:50,274,979-50,307,627 |
| PPP6R2 | ENSG00000100239 | 9557 | 9701 | 15.31320047 | 4.528518391 | chr22:50,343,331-50,444,374 |
| SBF1 | ENSG00000100241 | 6271 | 6305 | 26.98049869 | 10.66082063 | chr22:50,445,000-50,475,024 |
| ADM2 | ENSG00000128165 | 55726 | 79924 | 0.19730371 | 0.422955723 | chr22:50,481,556-50,486,440 |
| MIOX | ENSG00000100253 | 34875 | 55586 | 0.015527345 | 0.040565592 | chr22:50,486,876-50,490,648 |
| LMF2 | ENSG00000100258 | 60024 | 91289 | 7.769098179 | 3.618842841 | chr22:50,502,949-50,507,691 |
| NCAPH2 | ENSG00000025770 | 29516 | 29781 | 13.77045402 | 5.292933086 | chr22:50,508,216-50,523,472 |
| SCO2 | ENSG00000130489 | 9853 | 9997 | 13.15066773 | 5.760848405 | chr22:50,523,568-50,526,439 |
| TYMP | ENSG00000025708 | 1878 | 1890 | 0.94510355 | 1.906792374 | chr22:50,525,752-50,530,056 |
| ODF3B | ENSG00000177989 | 133981 | 440836 | 0.612032798 | 2.322889556 | chr22:50,530,409-50,532,579 |
| KLHDC7B | ENSG00000130487 | 76681 | 113730 | 0.034960576 | 0.057315639 | chr22:50,548,033-50,551,023 |
| SYCE3 | ENSG00000217442 | 151891 | 644186 | 0.450815943 | 0.823569282 | chr22:50,551,112-50,562,905 |
| CPT1B | ENSG00000205560 | 1366 | 1375 | 3.035423968 | 2.329872372 | chr22:50,568,862-50,578,417 |
| CHKB | ENSG00000100288 | 1112 | 1120 | 7.359764983 | 3.823618301 | chr22:50,578,949-50,582,999 |
| MAPK8IP2 | ENSG00000008735 | 23296 | 23542 | 60.60965588 | 26.39978049 | chr22:50,600,685-50,611,551 |
| ARSA | ENSG00000100299 | 407 | 410 | 5.644340376 | 2.767465025 | chr22:50,625,018-50,628,172 |
| SHANK3 | ENSG00000251322 | 59528 | 85358 | 15.82318406 | 12.10088919 | chr22:50,674,642-50,733,210 |
| ACR | ENSG00000100312 | 46 | 49 | 0.068265924 | 0.079773659 | chr22:50,738,196-50,745,334 |
| RABL2B | ENSG00000079974 | 10999 | 11158 | 2.99932746 | 1.086681557 | chr22:50,767,501-50,783,662 |

References

1. Kent WJ, Sugnet CW, Furey TS, Roskin KM, Pringle TH, Zahler AM, et al. The human genome browser at UCSC. Genome research. 2002;12(6):996-1006. Epub 2002/06/05. doi: 10.1101/gr.229102. PubMed PMID: 12045153; PubMed Central PMCID: PMCPMC186604.
