# Supplementary material for: Functional genomics analysis of Phelan-McDermid syndrome 22q13 region during human neurodevelopment
Source: PLoS One. 2019 Mar 15;14(3):e0213921. doi: 10.1371/journal.pone.0213921 (PMC6420160; doi:10.1371/journal.pone.0213921)
Supplement: S5 Table — Average brain region specific expression of each gene assessed is shown. Results of ANOVA testing is also displayed. Significant p-values are bolded. (AMY = amygdala, CBC = cerebellum, HIP = hippocampus, DLPFC = dorsolateral prefrontal cortex, VLPFC = ventrolateral prefrontal cortex, STR = striatum). (DOCX) [file pone.0213921.s005.docx]

| **Gene** |  |  |  |  |  |  |  |
| --- | --- | --- | --- | --- | --- | --- | --- |
|  | **Average expression per region** | | | | | |  |
|  | **AMY** | **CBC** | **DLPFC** | **VLPFC** | **HIP** | **STR** | **ANOVA p-value** |
| ***SHANK3*** | 17.916383 | 27.72675362 | 17.71278752 | 16.94101758 | 21.71830904 | 18.82812743 | **0.024033214** |
| ***ATNX10*** | 41.26779863 | 37.0037375 | 44.80037032 | 42.97920227 | 45.47487678 | 39.0065709 | 0.247876548 |
| ***MAPK8IP2*** | 63.37662096 | 62.99418485 | 70.94456716 | 68.82654369 | 62.56439704 | 39.3871891 | **0.000399707** |
| ***MLC1*** | 82.747505 | 47.45225092 | 46.4419014 | 51.68592231 | 72.4143823 | 54.65456443 | 0.088921645 |
| ***SULT4A1*** | 57.68490642 | 46.74587854 | 85.7907388 | 72.42424946 | 61.02446017 | 35.24227195 | **0.000848653** |

**S5 Table.** Expression data per region. Average brain region specific expression of each gene assessed is shown. Results of ANOVA testing is also displayed. Significant p-values are bolded. (AMY = amygdala, CBC= cerebellum, HIP = hippocampus, DLPFC = dorsolateral prefrontal cortex, VLPFC = ventrolateral prefrontal cortex, STR = striatum).
